# Catastrophic Outcomes in Free Tissue Transfer: A Six-Year Review of the NSQIP Database

**DOI:** 10.1155/2014/704206

**Published:** 2014-11-16

**Authors:** David W. Grant, Alexei Mlodinow, Jon P. Ver Halen, John Y. S. Kim

**Affiliations:** ^1^Division of Plastic and Reconstructive Surgery, Feinberg School of Medicine, Northwestern University, Chicago, IL 60611, USA; ^2^Division of Plastic and Reconstructive Surgery, Baptist Cancer Center, Vanderbilt Ingram Cancer Center, St. Jude Children's Research Hospital, Memphis, TN 38139, USA

## Abstract

*Background*. No studies report robust data on the national incidence and risk factors associated with catastrophic medical outcomes following free tissue transfer. *Methods*. The American College of Surgeons (ACS) multicenter, prospective National Surgical Quality Improvement Program (NSQIP) database was used to identify patients who underwent free tissue transfer between 2006 and 2011. Multivariable logistic regression was used for statistical analysis. *Results*. Over the 6-year study period 2,349 patients in the NSQIP database underwent a free tissue transfer procedure. One hundred and twenty-two patients had at least one catastrophic medical outcome (5.2%). These 122 patients had 151 catastrophic medical outcomes, including 93 postoperative respiratory failure events (4.0%), 14 pulmonary emboli (0.6%), 13 septic shock events (0.5%), 12 myocardial infarctions (0.5%), 6 cardiac arrests (0.3%), 4 strokes (0.2%), 1 coma (0.0%), and 8 deaths (0.3%). Total length of hospital stay was on average 14.7 days longer for patients who suffered a catastrophic medical complication (*P* < 0.001). Independent risk factors were identified. *Conclusions*. Free tissue transfer is a proven and safe technique. Catastrophic medical complications were infrequent but added significantly to length of hospital stay and patient morbidity.

## 1. Introduction

Since Seidenberg and colleagues reported the first free tissue transfer (FTT) in 1959 for esophageal reconstruction [1], and McLean and Buncke's first report in 1972 [2], free autogenous tissue transfer with microvascular anastomosis has become a safe and effective procedure in many reconstructive settings [3–6]. Its superior patient satisfaction has made it the gold standard in postoncologic breast reconstruction [7, 8], and it is requisite for the reconstruction of many oncologic, traumatic, and congenital defects. Yet while a great deal is known about surgical success and complication rates [3, 4, 9–14], much less is known about medical complications and their specific risk factors, despite the known negative effect of medical complications on recovery time, patient satisfaction, and healthcare system costs [5, 13, 15].

Among the most serious medical complications—rates of death, pulmonary embolism (PE), stroke, or myocardial infarction (MI)—there exists no benchmark to use when counseling patients about the risks of FTT surgery. Only data from individual surgeons operating at single institutions have been reported, and the data are both variable and incomplete [5, 6, 9, 12–22]. It is in this setting that a focused examination of catastrophic outcomes following FTT was undertaken.

The National Surgical Quality Improvement Program (NSQIP) database is a nationally validated, risk-adjusted surgical outcomes database, implemented to improve the quality of care delivered to surgical patients in the United States [23]. The robust cohort of patients undergoing FTT enables a high-powered retrospective analysis to be performed. Our focus was on identifying risk factors independently associated with catastrophic medical outcomes.

## 2. Patients and Methods

NSQIP was instituted by the American College of Surgeons in 2004 and provides a comprehensive database of 240 pre- and postoperative variables for over 1.3 million patients from over 250 institutions across the United States. Participant Use Data Files for 2006–2011 were downloaded from the ACS NSQIP website (http://www.acsnsqip.org/). The NSQIP database was retrospectively reviewed to obtain data on all patients undergoing free flap procedures with microvascular anastomosis between 2006 and 2011. The data collection methods for NSQIP have been previously described in detail [23–25].

Patients undergoing FTT procedures were identified by the presence of primary or concurrent current procedural terminology (CPT) codes 15756, 15757, 15758, 15842, 19364, 20969, 20970, 20972, 20973, 43496, and 49906, all of which refer to FTT procedures. Patients with incomplete demographic data were excluded.

Descriptive statistics, as well as complication profiles, were calculated for the study population. The primary endpoint was catastrophic medical outcomes (CMO), including the following complications: pulmonary embolism, postoperative respiratory failure (PRF, defined as failure to wean from mechanical ventilation for more than 48 hours and unplanned reintubation [26–28]), coma, stroke, cardiac arrest requiring CPR, myocardial infarction, septic shock, and death. Demographic data included age, body mass index (BMI), gender, and race. The only lifestyle variables included in the analysis were smoking status in the year before admission and whether the patient consumed more than 2 alcoholic drinks per day in the two weeks preceding admission. The following clinical characteristics were tracked: steroid use for chronic conditions, radiotherapy <90 days prior to surgery, chemotherapy <30 days prior to surgery, and a previous operation within 30 days prior to the index operation. The following comorbidities were tracked: diabetes mellitus (controlled with oral agents or insulin), hypertension requiring medication, dyspnea at rest or with moderate exertion, severe chronic obstructive pulmonary disease (COPD), dependence on mechanical ventilation, bleeding disorders, open wound/wound infection, SIRS/systemic sepsis/septic shock, previous cardiac surgery, rest pain/gangrene or revascularization procedure/amputation (peripheral vascular disease, PVD), transient ischemic attack (TIA) or stroke with/without neurological impairment (cerebrovascular disease, CVD), and disseminated cancer. The following surgical variables were tracked: total operation time, wound classification (clean, clean-contaminated, contaminated, and dirty/infected), and ASA classification. Length of total hospital stay was also tracked.

Statistical analyses were performed using SPSS version 20 (IBM Corp., Armonk, NY). Demographics, comorbidities, and outcomes were compared using chi-squared tests or Fisher's Exact Test for categorical variables and ANOVA for continuous variables. Significance level was set at *P* < 0.05. All preoperative variables with *P* < 0.2 and 10 or more occurrences in both groups upon univariate analysis were included in a binomial logistic regression.

## 3. Results

### 3.1. Study Population

In total 2,376 patients were identified who underwent FTT between 2006 and 2011. Nine patients (0.4%) had no gender listed and were deleted. An additional eighteen patients (0.8%) had height and/or weight data missing and were deleted. Of the remaining 2,349 patients, most were female (71.4%) and white (73.7%). Seventeen-point-three percent were active smokers in the year before surgery, and 3.2% consumed more than 2 alcoholic drinks per day in the 2 weeks before surgery. The most common comorbidity was hypertension requiring medication (34.3%). Ten percent of patients had open wound infections at the time of surgery, 8.7% of patients had diabetes, 5.6% of patients had dyspnea, and 5.1% had previous surgery within 30 days. All other comorbidities and clinical characteristics occurred in less than 5% of patients (Table 1).

### 3.2. Catastrophic Medical Outcomes

One hundred and twenty-two patients (5.2%) had a total of 151 CMO. One patient suffered a total of four catastrophic complications, 3 patients (0.1%) had three, 20 patients (0.9%) had two, and the remaining 98 patients (4.2%) had one, accounting for the 151 total CMO.

The most common CMO was postoperative respiratory failure, occurring in 93 patients (4.0%). Other events included 14 pulmonary emboli (0.6%), 13 episodes of septic shock (0.5%), 12 myocardial infarctions (0.5%), 6 cardiac arrests requiring CPR (0.3%), 4 cerebrovascular accidents (0.2%), 1 coma (0.0%), and 8 deaths (0.3%). Of these 8 patients who died, all were ASA class III or IV, only 1 patient had a clean case, 2 patients failed to wean from mechanical ventilation for >48 hours, 1 patient had a PE, 2 had cardiac arrests, 1 had a stroke, and no patients had MI, coma, or septic shock. A complete summary of the catastrophic events is presented in Table 2.

Considering clinical characteristics of the 122 patients who suffered at least 1 CMO, 35 patients (28.7%) were active smokers in the year before surgery, 8 patients (6.6%) had >2 alcoholic drinks/day in the 2 weeks preceding surgery, and 12 patients (9.8%) had a previous surgery within 30 days. Common comorbidities included hypertension (54.1%), diabetes (19.7%), open wound infection (21.3%), cerebrovascular disease (10.7%), dyspnea at rest or with moderate exertion (13.9%), disseminated cancer (9.0%), or a history of cardiac surgery (5.7%). A full summary of demographic and clinical characteristics of this cohort is presented in Table 3.

With respect to operative data, patients with an ASA classification of II or IV were twice as likely to have a CMO (89.9% versus 42.4%; *P* < 0.001) (Table 4). Similarly, patients with wound classification other than clean (i.e., clean-contaminated, contaminated, or dirty/infected) were significantly more likely to have CMO (*P* < 0.001). Length of stay for the CMO cohort was 21.7 days, versus 7.0 for the no-CMO patients (*P* < 0.001). Operative time was just under 2 hours longer in the group that had a catastrophic outcome (601.6 versus 489.3 minutes, *P* < 0.001). While infrequent, emergency cases were significantly associated with CMO (*P* = 0.043). Outpatient surgery was not associated with catastrophic outcomes (*P* = 0.146).

### 3.3. Binomial Regression

A binomial logistic regression analysis was performed using variables that met statistical requirements (*n* > 10 and *P* < 0.20) (Table 5). Five variables were found to be independently associated with CMO: bleeding disorder (OR 3.13, *P* = 0.003), CVD (OR 2.25, *P* = 0.021), wound classification other than clean (OR 2.20, *P* = 0.001), diabetes (OR 1.72, *P* = 0.048), ASA classification of class III or IV (OR 2.17, *P* = 0.004), and total operation time (OR 1.002, *P* < 0.001).

## 4. Discussion

Catastrophic medical outcomes (CMO) are generally defined as any perioperative sudden event that could potentially result in patient death or permanent disability within 30 days of surgery [29, 30]. CMO can occur from a number of specific events, including injury to vessels or nerves, retained devices, visceral perforation, and wrong-site surgery [31]. While the majority of these events are procedure-specific and are thus not tracked in NSQIP, it is possible to use the generic variables available in the dataset to identify CMO. These defined our primary endpoints and included the following complications: pulmonary embolism, postoperative respiratory failure (PRF, defined as failure to wean from mechanical ventilation for more than 48 hours and unplanned reintubation [26–28]), coma, stroke, cardiac arrest requiring CPR, myocardial infarction, septic shock, and death. Reconstructive microsurgery is unique in that it requires prolonged operative times, multiple surgical sites (i.e., flap donor and recipient sites), multiple surgical teams (in many cases), and often significant physiologic insult (e.g., composite head and neck resection, limb salvage after trauma). Thus, patients undergoing FTT are uniquely susceptible to CMO.

This study is the largest and most comprehensive examination to date of catastrophic medical outcomes following FTT. It establishes a benchmark for surgeons to use when counseling patients about the risks of undergoing FTT surgery. The overall 30-day mortality rate for our cohort was 0.3 percent, and the overall CMO rate was 5.2 percent. Pulmonary complications (PRF and PE) accounted for the majority of CMO (4.6 percent). While every CMO collected by NSQIP occurred at least once, nonpulmonary complications were rare (0.6 percent). These outcomes were independently related to the medical comorbidities of diabetes, CVD, and bleeding disorders. They were also independently related to the surgical indices of ASA class III or IV, and wound class other than clean (i.e., clean-contaminated, contaminated, and dirty/infected). An important (although unsurprising) additional result of this study was that CMO extended LOS by approximately 14.7 days (*P* < 0.001).

Advantages of the NSQIP database include a large cohort of 2,349 patients from geographically diverse inpatient and outpatient centers, with a large number of surgeons over 6 years. The above attributes make this paper an important contribution to the current literature on medical outcomes following FTT, which previously consisted primarily of single-institution, single-surgeon experiences, which did not completely evaluate catastrophic outcomes [5, 6, 9, 12–21, 32]. The data presented here is valid for generalization by institutions that do not themselves perform internal quality analyses and hence establishes a national benchmark for CMO following FTT. The limitations of NSQIP have been previously described [33–35]. Specific to this study, NSQIP only reports data for 30 days after surgery, leading to a likely underreporting of complications [36]. However, it is unlikely that CMO occur beyond 30 days postoperatively. The other limitation specific to this study is that some preoperative risk factors, like COPD, sepsis, or preoperative chemotherapy or radiotherapy, occurred too infrequently for inclusion in regression analysis, and so larger studies using NSQIP data from forthcoming years will aid in further defining independent risk factors for catastrophic outcomes. Finally, NSQIP does not track the specific type of free flap used for reconstruction, which can have implications for the type of CMO incurred with a given surgery [37]. Regardless of these limitations, the data presented in the paper is the most robust analysis of CMO after FTT.

Our study found an overall 30-day mortality of 0.3 percent, redemonstrating the safety of FTT. Mortality rates reported in the literature are quite variable, from as low as zero percent [13] to as high as 6.3 percent [9], likely reflecting the result of single-institution, single-surgeon studies. The data presented here indicate that the free flap mortality rate in the United States is less than estimates of overall surgical mortality in developed nations, at 0.4–0.8 percent [38], indicating that FTT is a safe surgical procedure. The overall CMO rate was 5.2 percent. Pulmonary complications (postoperative respiratory failure (PRF) and PE) occurred most frequently, at 4.6 percent. Other nonpulmonary complications were much rare at 0.6 percent.

In our study, PRF was the most common catastrophic outcome following FTT: it occurred in 93 patients (4.0 percent) and represents 61.6 percent of all 151 catastrophic outcomes. PRF is defined as failure to wean mechanical ventilation for >48 hours or unplanned reintubation [26–28]. PRF is one of the most serious pulmonary complications, and decreasing its incidence will have a large effect on outcomes and costs associated with FTT. PRF is known to increase morbidity and mortality [39] as well as to increase length of stay (LOS) and costs [40]. An analysis of Medicare data showed that PRF occurred in 73,136 patients (17.18 patients per 1000 hospitalized at-risk patients) over 2007–2009, had a mortality of 20.6 percent, and costed Medicare $1.96 billion US dollars [41]. PRF is thus a major burden on healthcare resources. Indeed, another study using the NSQIP database found PRF was associated with the largest attributable cost among the top 4 postoperative complications [40]. The Agency for Healthcare Research and Quality has identified PRF as 1 of 20 patient safety indicators to track healthcare outcomes [42], and in 2014 it will be included in the proposed rule for hospital value-based purchasing program for Medicare inpatient services [41]. Our report is the first to identify the national rate of PRF following FTT, at four percent. Adequate understanding of its risk factors, prevention, and treatment are thus important areas for future investigation.

Pulmonary embolism was the second most common CMO, occurring in 0.6 percent of all patients undergoing FTT and accounting for 9.3 percent of all 151 CMO. The 14 cases of PE occurred throughout the 30 days of available data (range POD 1–24; data not shown) and thus were not clustered in the immediate postoperative period. Rates of PE reported in the literature vary from near zero percent [13, 43] to 3.8 percent [12], again likely reflecting single-institution, single-surgeon studies. Our data again provide an important benchmark of the national multi-institutional rate of PE after FTT and establish PE as the second most common CMO following FTT. In addition, DVT or thrombophlebitis occurred in 6 of the 122 patients (4.9 percent) who suffered a CMO, compared to 11 of the 2,227 patients who did not (0.5 percent) (*P* < 0.001, Fisher's Exact Test; data not shown), suggesting insufficient DVT prophylaxis among patients who suffer CMO following FTT. Indeed, studies of anticoagulation in head and neck FTT patients reveal that less than half of patients achieve therapeutic anticoagulation [19]. Taken together, these findings underscore the importance of adequate VTE prophylaxis in FTT patients. Similar to PRF, postoperative PE or DVT occurred in 130,927 patients from 2007 to 2009 and costed Medicare $1.42 billion in excess costs. For these reasons, it is also included in the proposed rule for hospital value-based purchasing program for Medicare inpatient services [41].

While the overall CMO rate was low at 5.2 percent, an important finding of this study was that such outcomes were significantly associated with a longer length of total hospital stay (LOS), by an average of just over 2 weeks (*P* < 0.001). Thus, while FTT is in general safe, the financial impact of CMO is tremendous. We did not examine this directly, but Fischer and colleagues recently reported that medical complications added about $350,000 of direct costs following FTT for breast reconstruction [13]. Their medical complications were less severe, adding an average of only 1.72 days to their patients' hospital stay, contrasting to 14.7 days added for patients who suffered a CMO in the present report. Preventive strategies targeted at these CMO could thus result in substantial healthcare savings and further optimize the safety of these procedures.

Our regression analysis identified preoperative risk factors that independently predicted CMO, including bleeding disorders, diabetes, and cerebrovascular disease. Wound classification of anything* other than* a clean case (i.e., clean-contaminated, contaminated, dirty/infected) and ASA class of III or IV also independently predicted CMO. Total operation time was also an independent predictor, as reported elsewhere [44]. While the impact of each individual minute was small (OR 1.002), it is understandable how increased operative times of 1-2 hours (or more) could result in clinically significant CMO. The authors thus argue that every effort should be made to maximize efficiency in the operating room and minimize operative time (without sacrificing patient safety or best medical practice).

A number of factors associated with adverse events are well described in the literature, but their association with CMO merits special mention. ASA scores have been utilized as a substitute for overall physical fitness [45]. The potential subjectivity of this value has been a topic of controversy in outcomes studies, yet these results continue to demonstrate its association with outcomes. Consistent with these findings, our study found a significant association between ASA class III or IV and CMO. Diabetes mellitus is associated with a long list of postoperative surgical and medical complications, including stroke, myocardial infarction, and wound infection [46, 47]. Such findings argue for thorough preoperative optimization of patients before undergoing FTT, particularly with regard to ASA class. We have further adopted a strict protocol for evaluation of diabetic patients. Patients with hemoglobin A1C levels above 7% undergo preoperative medical evaluation to ensure that they have an adequate regimen for diabetic control. All patients abstain from oral hypoglycemic agents the day of surgery but receive one-half of their regular insulin dose. Postoperatively, all patients are started on intravenous insulin drips with continuous titration and resume oral hypoglycemics (if tolerating oral medications and diet) on postoperative day one. Finally, a history of cerebrovascular disease (CVD) has previously been associated with adverse events, presumably owing to issues with airway protection and postoperative mobilization [48, 49]. While a history of CVD is not routinely recognized in single-center outcomes studies as a factor associated with AEs, it has repeatedly appeared in large-database studies, presumably owing to the power of these studies to find significant associations in large numbers of patients. Pursuant to this finding, we routinely request speech therapy evaluations prior to oral intake in patients with difficult speaking or swallowing and rigorously follow positioning, hygiene, physical and occupational therapy, and venous thromboembolic prevention protocols for patients with impaired mobility.

An association between contaminated wounds and adverse events after surgery is well documented. A 2011 study by Lee et al. showed a direct relationship between wound contamination with oral flora and postoperative wound infection, while other studies have found that patients undergoing mandibulectomy, glossectomy, tonsillectomy, and laryngectomy had a significant risk for postoperative pneumonia compared to those undergoing pharyngectomy and esophagectomy, further increasing the length of hospitalization and mortality [50, 51]. Such findings are consistent with the current study, showing increased CMO in the setting of contaminated surgical wounds, and, in cases of head and neck surgery, posit a causative mechanism. Given that contaminated wound status is associated with CMO, what can be done about it? The role of prophylactic antibiotics in preventing surgical site infection in head and neck procedures has been extensively studied at single institutions [52–55]. Studies in the general surgery literature suggest that 24 hours of antibiotic therapy is an adequate treatment period [56, 57]. In the head and neck literature, there has been some evaluation of optimal duration of antibiotic usage, but there is a lack of multi-institutional studies evaluating this. The use of prophylactic antibiotics in clean-contaminated cases has been shown to reduce the risk of wound infection from ranges of 30–80% to 22–47%. A prospective, randomized trial by Righi et al. of 192 patients undergoing aerodigestive or laryngeal surgery through a cervical skin incision was divided into groups receiving either 1 or 3 days of antibiotic prophylaxis. The study failed to show a significant difference in the rate of wound infection, regardless of antibiotic duration. Finally, the principals of debridement of all necrotic or devitalized material and timely surgical intervention before significant infection can be established (as with extremity salvage or sternal wound infection), or of delaying reconstruction until wounds are free of surgical infection are considerations highlighted by our study.

Of the presurgical comorbidities, a history of bleeding disorders was independently associated with catastrophic outcomes and was found to have the highest associated odds ratio (OR 3.13, *P* = 0.003). Bleeding disorders are defined by NSQIP as “any condition that places the patient at risk for excessive bleeding requiring hospitalization due to a deficiency of blood clotting elements (e.g., vitamin K deficiency, hemophilias, thrombocytopenia, chronic anticoagulation therapy that has not been discontinued prior to surgery). Patients are not included who are on chronic aspirin therapy.” Few papers describe the implications of hematologic disorders in free tissue transfer [58–61]. These patients typically suffer postoperative bleeding at the sites of vascular anastomoses or donor site and reoperation [58–60]. Given the focus of our study, we did not include these complications for analysis, but we did identify that, in addition to well-known surgical complications, bleeding disorders are also independent predictors of catastrophic medical complications following FTT. A major limitation of the NSQIP database is that further information regarding the type and nature of a patient's bleeding disorder is not available. Possible explanations are that patients with hematological disease often have other medical problems [58] which could complicate their surgery, that is, hematological malignancies causing thrombocytopenia and/or pancytopenias, predisposing to infection, sepsis, and death [58, 62]. NSQIP also categorizes patients who have not withheld their chronic anticoagulation before surgery as having a bleeding disorder. However, most microsurgeons report the use of anticoagulants in their routine practice [10, 19], although this is controversial [22, 63]. While most discussions of anticoagulation and bleeding disorders focus on flap loss, our study adds an important contribution that bleeding disorders are also associated with CMO. Further research focusing on patients with bleeding disorders, with reference to specific disorders, is needed to clarify this relationship. It has also been estimated that over 95% of clinical hematologic abnormalities can be screened by simply asking the patient, “Have you, or anyone in your family, had and history of bleeding or clotting disorders?” [64]. For patients who respond affirmatively to this question, we routinely send patients for preoperative hematology evaluations. While this may seem to be an onerous burden to patients, physicians, and payers alike, data linking hematologic abnormalities to both free flap loss and now CMO after FTT makes this evaluation worthwhile.

Of note, patients with pulmonary comorbidities before surgery, including dependence on mechanical ventilation and dyspnea at rest or with moderate exertion, were all significantly associated with CMO on univariate analysis. Seventeen of the 122 patients who suffered a CMO had dyspnea at rest or with moderate exertion. Dyspnea was included in the logistic regression but was found not to be an independent predictor of catastrophic outcomes (*P* = 0.304). Severe COPD and ventilator dependence were too infrequent to be included in regression analysis, so we cannot determine if they are independently associated with catastrophic outcomes. Other studies have found that COPD is independently associated with medical complications (ranging from minor to severe) following FTT, with odds ratios as high as 17.2 [13]. Considering that the majority (4.6 of the total 5.2 percent) of CMO following FTT were pulmonary in nature, preoperative optimization strategies are an excellent target for intervention. These include at the minimum preoperative PA and lateral chest X-rays but more reasonably include a formal respiratory evaluation with a pulmonary specialist and perhaps either pulmonary function tests or CT scans based on the severity of the patient's disease. Based on the results of this study, we have developed same-day extubation protocols for patients undergoing FTT and aggressive postoperative pulmonary toilet (both on and off the ventilator) and are a part of our routine management. While it was not found to be independently associated with CMO after FTT, smoking is a well-documented predictor of surgical complications and surgical failure following reconstructive surgery [17, 32, 35]. The authors still consider it prudent to counsel patients to quit several months before any major surgery [65, 66] and now routinely request preoperative consultations for patients with any smoking history greater than 15 pack-years and for all patients with a smoking history associated with any pulmonary comorbidity.

## 5. Conclusion

This is the largest single clinical study examining catastrophic outcomes following FTT. The data suggest that FTT is safe, with a 30-day mortality of 0.3 percent. Overall, catastrophic medical complications occurred in 5.2 percent of patients and were associated with a two-week delay in hospital discharge on average. Independent risk factors for catastrophic medical outcomes included wound classification of clean-contaminated, contaminated, or dirty/infected, ASA classification of class III or IV, bleeding disorders, diabetes, and CVD. The results presented in the study provide the microsurgeon with useful risk-stratifying criteria to improve patient outcomes and indicate directions to reduce healthcare costs.

## Figures and Tables

**Table 1:** Population demographics: all free tissue transfers in NSQIP database from 2006 to 2011.

	All patients	
	(*n* = 2349)	
	*n*	%
Age (years, mean ± SD)	54.3 ± 12.9	
BMI (kg/m^2^, mean ± SD)	27.9 ± 6.3	
Sex		
Female	1678	71.4%
Male	671	28.6%
Race (%)		
White	1732	73.7%
Black	244	10.4%
Asian	61	2.6%
Other	312	13.3%
Clinical characteristics		
Active smoker within one year	406	17.3%
EtOH > 2 drinks/day	76	3.2%
Steroid use for chronic condition	38	1.6%
Radiotherapy <90 days	36	1.5%
Chemotherapy <30 days	94	4.0%
Previous OP <30 days	121	5.1%
Comorbidities		
Diabetes mellitus with oral agents or insulin	204	8.7%
Hypertension	805	34.3%
Dyspnea	132	5.6%
Bleeding disorders	51	2.2%
COPD	66	2.8%
Open wound/wound infection	235	10.0%
Disseminated cancer	88	3.7%
Number of patients with PVD	25	1.1%
Number of patients with CVD	66	2.8%

∗ denotes significant value, *P* < 0.05.

BMI: body mass index; EtOH: alcohol use; OP: operation; COPD: chronic obstructive pulmonary disease; PVD: peripheral vascular disease; CVD: cerebrovascular disease.

**Table 2:** Catastrophic outcomes.

Postoperative outcome	Total number of patients	% of all patients (*n* = 2349)	% of catastrophic outcomes (*n* = 122)
4 outcomes	1	0.0%	0.8%
3 outcomes	3	0.1%	2.5%
2 outcomes	20	0.9%	16.4%
1 outcome	98	4.2%	80.3%
Total	**122**		
Type of outcome			
Pulmonary embolism	14	0.6%	9.3%
PRF	93	4.0%	61.6%
Coma	1	0.0%	0.7%
Stroke	4	0.2%	2.6%
Cardiac arrest requiring CPR	6	0.3%	4.0%
Myocardial infarction	12	0.5%	7.9%
Septic shock	13	0.5%	8.6%
Death	8	0.3%	5.3%
Total	**151**		
Overall catastrophic complications^*^	122	5.2%	

^*^Patients can have more than one catastrophic complication.

PRF: prolonged respiratory failure; CPR: cardiopulmonary resuscitation.

**Table 3:** Population demographics, stratified by catastrophic outcome.

	Catastrophic outcome		No catastrophic outcome			*P* value
	*n* = 122		*n* = 2,227			
	*n*	%	*n*	%		
Age (years, mean ± SD)	61.0 ± 14.3		53.9 ± 12.7			0.000^*^
BMI (kg/m^2^, mean ± SD)	26.4 ± 6.5		27.9 ± 6.3			0.007^*^
Sex						
Male	73	59.8%	598	26.9%		0.000^*^
Female	49	40.2%	1629	73.1%		
Race (%)						
White	93	76.2%	1639	73.6%		
Black	6	4.9%	238	10.7%		
Asian	1	0.8%	60	2.7%		
Other	22	18.0%	290	13.0%		
Clinical characteristics						
Active smoker within one year	35	28.7%	371	16.7%		0.001^*^
EtOH > 2 drinks/day in preceding 2 wks	8	6.6%	68	3.1%	§	0.057
Steroid use	5	4.1%	33	1.5%	§	0.044^*^
Radiotherapy <90 days	7	5.7%	29	1.3%	§	0.002^*^
Chemotherapy <30 days	4	3.3%	90	4.0%	§	1.000
Previous OP <30 days	12	9.8%	109	4.9%		0.016^*^
Comorbidities						
Diabetes	24	19.7%	180	8.1%		0.000^*^
Hypertension	66	54.1%	739	33.2%		0.000^*^
Dyspnea	17	13.9%	115	5.2%		0.000^*^
Bleeding disorder	12	9.8%	39	1.8%	§	0.000^*^
Ventilator dependent status preop	3	2.5%	3	0.1%	§	0.002^*^
History of severe COPD	7	5.7%	59	2.6%	§	0.080
Systemic sepsis	8	6.6%	42	1.9%		0.004^*^
Open wound infection	26	21.3%	209	9.4%		0.000^*^
Disseminated cancer	11	9.0%	77	3.5%	§	0.005^*^
History of cardiac surgery	7	5.7%	32	1.4%	§	0.003^*^
Peripheral vascular disease	2	1.6%	23	1.0%	§	0.376
Cerebrovascular disease	13	10.7%	53	2.4%	§	0.000^*^

∗ denotes significant value, *P* < 0.05.

^§^Fisher's Exact Test; all others Pearson chi-square.

BMI = body mass index; EtOH = alcohol consumption; OP = operation; COPD = chronic obstructive pulmonary disease.

**Table 4:** Operative data.

	Catastrophic outcome		No catastrophic outcome			*P* value
	*n* = 122		*n* = 2227			
	*n*	%	*n*	%		
Outpatient setting	2	1.6%	97	4.4%		0.146
Emergency case	4	3.3%	22	1.0%	§	0.043^*^
ASA classification III or IV	98	89.9%	945	42.4%		0.000^*^
Wound classification 2, 3, or 4	86	70.5%	685	30.8%		0.000^*^
Operative time (minutes ± standard deviation)	601.6 ± 264.6		489.3 ± 217.0			0.000^*^
Length of total hospital stay (days ± standard deviation)	21.7 ± 17.0		7.0 ± 7.6			0.000^*^

∗ denotes significant value, *P* < 0.05.

^§^Fisher's Exact Test; all others Pearson chi-square.

ASA: American Society of Anesthesiologists.

**Table 5:** Binomial logistic regression.

Predictor	*P* value	Odds ratio	95% confidence interval	
Patient age	0.427	1.01	0.99	1.02
Male gender	0.116	1.43	0.92	2.23
BMI	0.135	0.97	0.94	1.01
Active smoker within one year	0.473	1.19	0.74	1.89
Previous OP <30 days	0.146	1.67	0.84	3.32
Wound classification 2, 3, or 4^*^	**0.001**	**2.20**	1.36	3.57
ASA classification III or IV^*^	**0.004**	**2.17**	1.27	3.69
Diabetes^*^	**0.048**	**1.72**	1.01	2.93
Hypertension	0.131	1.40	0.91	2.16
Dyspnea	0.304	1.37	0.75	2.51
Bleeding disorder^*^	**0.003**	**3.13**	1.49	6.60
Open wound infection	0.275	1.33	0.80	2.22
Disseminated cancer	0.876	1.06	0.52	2.15
Cerebrovascular disease^*^	**0.021**	**2.25**	1.13	4.49
Total operation time^*^	**0.000**	**1.002**	1.00	1.00

∗ denotes significant value, *P* < 0.05.

H-L statistic: 0.736.

c-statistic: 0.815.

Criteria for inclusion into binomial logistic regression included *P* < 0.2 on univariate analysis and *n* > 10. BMI: body mass index; OP: operation; ASA: American Society of Anesthesiologists.
